# Nanostructured Lipid Carriers: A Groundbreaking Approach for Transdermal Drug Delivery

**DOI:** 10.34172/apb.2020.021

**Published:** 2020-02-18

**Authors:** Iti Chauhan, Mohd Yasir, Madhu Verma, Alok Pratap Singh

**Affiliations:** ^1^Department of Pharmaceutics, I.T.S College of Pharmacy, Muradnagar, Ghaziabad- 201206, Uttar Pradesh, India.; ^2^Department of Pharmacy, College of Health Science, Arsi University, Asella, Oromia Region, Ethiopia.

**Keywords:** Nanostructure lipid carrier, Lipid, Topical, Skin

## Abstract

Nanostructured lipid carriers (NLCs) are novel pharmaceutical formulations which are composed of physiological and biocompatible lipids, surfactants and co-surfactants. Over time, as a second generation lipid nanocarrier NLC has emerged as an alternative to first generation nanoparticles. This review article highlights the structure, composition, various formulation methodologies, and characterization of NLCs which are prerequisites in formulating a stable drug delivery system. NLCs hold an eminent potential in pharmaceuticals and cosmetics market because of extensive beneficial effects like skin hydration, occlusion, enhanced bioavailability, and skin targeting. This article aims to evoke an interest in the current state of art NLC by discussing their promising assistance in topical drug delivery system. The key attributes of NLC that make them a promising drug delivery system are ease of preparation, biocompatibility, the feasibility of scale up, non-toxicity, improved drug loading, and stability.

## Introduction

Currently, the field of nanotechnology is intensely exploited for drug delivery technology for passive and active targeting via various routes of administration. The application of nanotechnology in transdermal and topical drug delivery has proclaimed a new domain in the delivery of pharmaceuticals via the skin. Nanoparticles are defined as colloidal particulate systems having dimensions between 10-1000 nm.^1^ Solid lipid nanoparticles (SLNs) were conceptualized in early 1990s by Professor R.H. Müller (Germany) and Professor M. Gasco (Italy) as a novel formulation possessing several advantages e.g. the use of biocompatible lipids, least use of organic solvents during formulation, high *in vivo* stability and broad application spectrum.^2^ SLNs are colloidal particles prepared from solid lipids (solid at room temperature and body temperature), surfactants, active ingredient and water. Still, SLNs experience certain limitations like poor drug loading capacity, unpredictable gelation tendency, polymorphic transitions and drug leakage during storage.^1-3^

Nanostructured lipid carriers (NLCs) spring up as second generation of lipid nanoparticles to overcome the shortcomings of first generation i.e. SLNs. Biodegradable and compatible lipids (solid and liquid) and emulsifiers are used for the preparation of NLCs. Liquid lipids (oil) incorporation causes structural imperfections of solid lipids leading to a less ordered crystalline arrangement which avert drug leakage and furnish a high drug load.^2,4^ In last few years, NLCs have gained attention of researchers as an alternative of SLNs, polymeric nanoparticles, emulsions, microparticles, liposomes etc.^5^ These nanocarriers possess the utility in delivery of hydrophilic as well as lipophilic drugs. NLCs have emerged as a promising carrier system for the delivery of pharmaceuticals via oral, parenteral, ocular, pulmonary, topical, and transdermal route. Recently, NLCs are also being exploited in brain targeting, chemotherapy, gene therapy, food industry and delivery of cosmeceuticals and nutraceuticals.^5^ Table 1 mentions the advantages and disadvantages of NLC.

**Table 1:** Advantages and disadvantages of Nanostructured Lipid Carriers^5-7^

**Advantages**	**Disadvantages**
More loading capacity for some drugs	Cytotoxic effects related to the nature of lipid matrix and concentration
Less water in the dispersion	Irritation and sensitizing action of surfactants
Prevent or minimize the drug expulsion during storage	Application and efficiency in case of protein and peptide drugs and gene delivery systems still need to be exploited
Control and targeted drug release	Stability of Lipids
Feasibilities of loading both lipophilic and hydrophilic drugs	-
Use of biodegradable and biocompatible lipids	-
Avoid organic solvents	-
More affordable (less expensive than polymeric/surfactant based carriers	-
Easier to qualify, validate and gain regulatory approval	-
Better physical stability	-
Ease of preparation and scale-up	-
Improve benefit/risk ratio	-
Increase of skin hydration and elasticity	-
Small size ensures close contact with the stratum corneum	-
Enhanced stability of drugs	-

### Types of NLC

**Figure 1:**
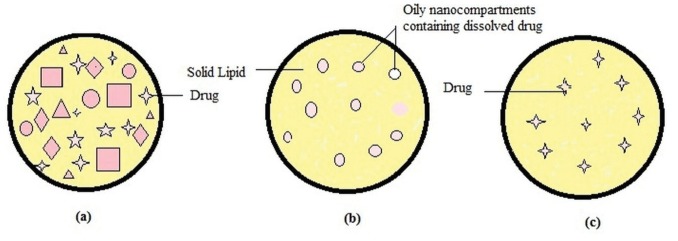
Types of nanostructure lipid carrier.^7^

Depending on the location of incorporated drug moieties in NLC, following three types of morphological models (Figure 1) has been proposed:

#### NLC type I (imperfect crystal model)

Imperfect crystal type NLC consists of a highly disordered matrix with many voids and spaces which can accommodate more drug molecules in amorphous clusters. These imperfections in the crystal order are acquired by mixing solid lipids with adequate amount of liquid lipids (oils). Due to varying chain length of fatty acids and the mixture of mono-, di-, and triacylglycerols, the matrix of NLC is not able to form a highly ordered structure. Mixing spatially different lipids increases drug payload capacity however this model offers minimum entrapment efficiency.^8,9^

#### NLC type II (multiple type)

Multiple type NLC is oil/ lipid / water type. Lipophilic drugs are more soluble in liquid lipids than solid lipids. This idea leads to the development of multiple type NLC using high liquid lipid content. Oil moieties, at low concentrations, are effectively dispersed in the lipid matrix. The addition of oil beyond its solubility induces phase separation forming small nano compartments of oil encircled in the solid matrix. Type II model offer advantages like high drug entrapment efficiency, controlled drug release and minimized drug leakage.^9,10^

#### NLC type III (amorphous model)

Amorphoustype NLC is formulated by carefully mixing lipids in such a way as to minimize the drug leakage due to process of crystallization. Specific lipids such as hydroxyl octacosanyl, hydroxyl stearate, isopropyl myristate or dibutyl adipate form solid yet non-crystalline particles. The lipid matrix exists in a homogenous amorphous state.^8-10^

### Excipients used in formulating NLC

Generally, NLCs are composed of lipid (s) (solid as well as liquid), surfactant(s), organic solvent and other agents like counter-ions and surface modifiers. Table 2 enlists some of the excipients used for formulating NLC.

**Table 2:** Excipients used in formulating NLC^6,7,11^

**Ingredient**	**Examples**
Solid lipid	Bees wax, Caranauba wax 2442, Stearic acid, Cetyl Palmitate, Apifil®, Cutina CP®, Dynasan® 116, Dynasan®118,Precifac ATO, Compritol®888 ATO, Elfacos® C 26, Imwitor 900®,Precirol® ATO 5, tristearin, cholesterol, Palmitic acid
Liquid lipids (oils)	Cetiol V, Miglyol® 812, Castor oil, oleic acid, Davana oil, Palm oil, Olive oil, Isodecyl oleate, Paraffin oil, propylene glycol dicaprylocaprate, linoleic acid, decanoic acid, Argan oil, coconut oil
Emulsifying agents	Pluronic® F68 (poloxamer 188), Pluronic® F127 (poloxamer 407), Tween 20, Tween 40, Tween 80,polyvinyl alcohol, Solutol® HS15, trehalose, sodium deoxycholate, Sodium glycocholate, sodium oleate,polyglycerol methyl glucose distearate, Tego®Care 450, Tween™80, Maquat® SC 18Maquat® BTMC-85%, Egg lecithin, soya lecithin, phosphatidylcholines, phosphatidylethanolamines, Gelucire® 50/13, Miranol ultra 32
Counter-ions	Sodium hexadecyl phosphate, Monodecyl phosphate, Mono hexadecyl phosphate, Mono octyl phosphate, Dextran sulphate sodium salt, Hydrolysed and polymerised epoxidised soybean oil

### Lipids

The primary component of nanostructure lipid carriers that govern drug loading capacity, prolong action and stability of the formulations is lipid. Solid lipids like fatty acids, triglyceride, diglyceride, monoglyceride, steroids and waxes have been used for formulating NLC.^12^ Physiologically acceptable, biodegradable, non-toxic and generally-recognized-as-safe (GRAS) status lipids are preferred for preparation of lipid nanoparticles.^10^

Choice of suitable lipids is essential preceding their utilization in preparation of nanoparticulate carriers. The type and structure of lipid affects various characteristics of nanocarriers. Practically, solubility or evident partition coefficient of bioactives in the lipid has been suggested as the best fitting criteria for choosing a suitable lipid. The solubility of the drug molecules in lipid is interpretative as it affects drug loading and encapsulation efficiency.^10^ Degree of crystallization of various lipids employed also affect drug entrapment and loading, size and charge, and efficacy.^12^

On account of higher viscosity of dispersed phase, because of higher melting lipids, the average particle size of nano dispersion increases. Shape of lipid crystals, lipid hydrophilicity, variation in composition are additional lipid related parameters that may influence quality of NLC. It has been found that a 5- 10% hike in lipid amount leads to larger particle size.^13^

### Surfactants

The type and concentrations of surfactant exert influence on quality and efficacy of NLC. It has been found that toxicity, physical stability and crystallinity of NLC are greatly influenced by choice of surfactant.^14^ Surfactant systems also have an impact on extent of drug dissolution and drug permeability. Surfactants are chosen based on of route of administration, hydrophilic-lipophilic balance (HLB) value, effect on particle size and lipid modification. Surface active agents (emulsifiers) are adsorbed on the interface where they reduce the tension between lipid and aqueous phases because of their amphipathic nature.^5^ During the formulation of NLC, crystallization of colloid particles goes along with solidification, but the surface area of particle increase remarkably during crystallization so that the whole system become unstable. Hence, surfactant is a requisite to improve interface quality of nanoparticles to attain stability.^15^ Modifying the surfactant system compositions may govern the miscibility of chemical components in NLCs, and hence the stability.^14^

Required HLB (rHLB) plays an important role while selecting suitable type and amount of surfactant for NLC formulation.^16^ rHLB of lipids and lipid matrix is determined to calculate the amount of emulsifiers to be added in formulation. The rHLB value for lipid is the HLB value of emulsifier which is necessary for appropriate emulsification i.e. reduction of interfacial tension between oil and water phase. This also assists in achieving a stable nano system and small particle size of NLCs.^17,18^ By determining rHLB a right combination of emulsifiers with least concentration can be employed for formulation. rHLB for lipids (solid and liquid) and lipid matrix is calculated experimentally by dispersing in blends of surfactant with different HLB values. The mixture is put through high pressure homogenization and analyzed for least particle size.^16,18,19^

### Other ingredients

Organic salts and ionic polymers may be employed as counter-ions in formulation of nano structure carriers to overcome the challenge of encapsulating water soluble drug molecules. Surface-modifiers are another category of excipients used in formulation of NLC to minimize their phagocytic uptake by the macrophages in reticuloendothelial system (RES). Lipid particles are coated with hydrophilic polymers like PEG, poloxamines or poloxamers to increase the residence time of drug molecules in systemic circulation. Surface modification may offer other advantages like enhanced physical stability and biocompatibility, drug targeting, increased transport across epithelium.^10,20^

### Methods of preparing NLC

#### High-pressure homogenization technique

This technique is powerful and reliable for the commercial-scale production of NLCs. High pressure used in homogenization technique makes it possible to avoid use of organic solvents in preparations and render them eco friendly. Additionally high-pressure homogenization is easy to scale up and an attractive technique being used in the manufacturing of pharmaceuticals and cosmetics for topical application.^21^ Hot homogenisation is performed at elevated temperature and cold homogenization is done below room temperature. Active ingredient is dissolved or dispersed in the molten lipid before to the high pressure homogenization, in both approaches. High pressure (100–2000 bar) moves the fluid in the narrow gap in homogenizer.

#### Hot homogenization

In this approach homogenization is conducted at elevated temperature. The solid lipids are melted at a temperature above 5-10°C above their melting point. A dispersion is obtained by adding liquid lipid and drug to be encapsulated. The mixture is dispersed in aqueous solution of surfactant (s) heated to same temperature by high shear mixing device and leads to formation of pre emulsion. The pre-emulsion is introduced in high pressure homogenizer at controlled temperature. Generally 3 to 5 cycles at 500-1500 bar are sufficient for homogenization. The lipid recrystallizes and causes formation of nanoparticles as nanoemulsion is gradually cooled down. Employment of high temperature during the process may lead to degradation of heat sensitive ingredients. Another problem which may arise is reduction in emulsifying capacity of surfactants due to high temperature as surfactants have cloud point lower than 85°C. This may induce instability to nanocarriers.^4,6,21-23^

#### Cold homogenisation

In this technique lipid melt containing active agent is rapidly cooled to being solidify using liquid nitrogen or dry ice, then milled and ground before being dispersed in cold surfactant phase and subsequently homogenized at room temperature. Pressure used in cold process is higher i.e. 5-10 cycles of 1500 bar. This approach minimizes the thermal exposure of drug and well suited for thermolabile drugs. Improved drug entrapment efficiency and uniform distribution of drug within the lipid are other benefits of the method. However it results in nanoparticles of more variable sizes.^4,22-24^

### Solvent-emulsification evaporation method

In this method, the lipids (solid lipid + liquid lipid) along with drug are dissolved in a water immiscible organic solvent (cyclohexane, chloroform).^25^ The obtained mixture is dispersed into aqueous solution of emulsifiers producing an o/w emulsion. Evaporation under reduced pressure is employed to remove solvent from the emulsion. Evaporation leads to the dispersion of nanoparticles in the aqueous phase (by lipid precipitation in the aqueous medium). This method avoids any thermal stress, but usage of organic solvent is a disadvantage. Particle size can vary from 30-100 nm according to the solid lipid and surfactant.^6,24^

### Solvent-emulsification diffusion method

In this technique, solvent and water are mutually saturated to maintain initial thermodynamic equilibrium.

Afterwards, the lipids and drug is dissolved in the water-saturated solvent. Solvent containing drug and lipids are emulsified in a solvent-saturated aqueous emulsifier solution by a homogenizer to form an o/w emulsion. The lipid nanoparticles precipitate after dilution with excess water (ratio: 1:5–1:10) due to diffusion of the organic solvent from the emulsion droplets to the continuous phase. The solvent can be removed by ultrafiltration or lyophilisation. Solvent diffusion is more innovative and most of the solvent employed show a better safety profile compared to volatile solvents.^25^

### Microemulsion method

In this approach, the solid lipid is melted, followed by addition of liquid lipid and solubilization of drug in the subsequent mixture. Separately, a mixture of emulsifier, co-emulsifier and water is heated at same temperature. Both the lipid and the aqueous phase are mixed in appropriate ratios and gently stirred to produce thermodynamically stable oil in water hot microemulsion. The hot microemulsion is quickly dispersed into an excess of chilled water (0-4°C) with vigorous stirring. The dilution causes the breakdown of microemulsion into a nanoemulsion with ultrafine particles. The ratio of the hot microemulsion to cold water usually lies in the range of 1:10 to 1:50. As the microemulsion is diluted by cold water, the internal lipid droplets recrystallize to form nanosize carriers. The size of the nanoparticles depends on the droplet size of microemulsion and temperature difference between microemulsion and ice water. Rapid cooling and hence solidification can prevent the aggregation of particles and lead to production of smaller particles.^26^ NLC dispersions formed by this method contained large quantity of particles in the micron range; therefore, in this condition, the time of stirring, percentage of lipids, and amount of drug were optimized in order to obtain the appropriate size and higher entrapment efficiency. The method does not require any special equipment or energy for production of NLC; hence it is simple to commercially scale up the technique.^22,27,28^

### Double emulsion technique

This method is mainly used for the production of lipid nanoparticles loaded with hydrophilic drugs. This technique overcomes the problem of escapism of water soluble moiety in aqueous phase from oily phase as investigated in microemulsion method.^29^ In this method, drug is firstly dissolved in aqueous solvent (inner aqueous phase) and then is dispersed in lipid phase (Molten solid lipid + liquid lipid+ lipophilic surfactant+ lipophilic active moiety) to produce primary emulsion (w/o). Both lipid and the aqueous phase are maintained at same temperature. Stabilizer prevents loss of drug to the external phase during solvent evaporation. Afterwards, primary emulsion is dispersed into a large volume of surfactant aqueous solution followed by sonication to form a double emulsion (w/o/w). The lipid nanoparticles are then purified by ultrafiltration or solvent evaporation.^24,30^

### Solvent Injection technique

It is a viable new technique to manufacture lipid nanoparticles. In this technique, lipids are solubilized in water-miscible solvent (e.g., acetone, methanol, ethanol, isopropyl alcohol) or water-soluble solvent mixture and then rapidly injected into aqueous surfactant solution under continuous stirring. Resultant dispersion is filtered in order to eliminate excess lipid.^31^ The technique relies on rapid diffusion of the solvent over the solvent–lipid interfaced with the aqueous phase.^32^ The particle size of nanocarriers depends on diffusion rate of the organic solvent through the lipid-solvent interface. This method offers the advantage of easy handling, efficiency, versatility, no employment of technical equipment (e.g., high-pressure homogenizer) and use of approved organic solvents.^31^

### High shear homogenization and ultrasonication

One of the methods for the production of NLCs is high shear homogenization or ultrasonification. These dispersing techniques employ devices to prepare nanocarriers. Solid and liquid lipid is melted and dispersed in an aqueous surfactant solution under high shear homogenization or ultrasonication resulting in formation of nanodispersion.^33,34^ Intense shear forces necessary for the nano-emulsification are generated by ultrasonic cavitation which produces violently and asymmetrically imploding vacuum bubbles and break up particles down to the nanometer scale.^35^

Probe-type ultrasonication produce desired effects like homogenization, dispersion, deagglomeration, milling and emulsification.^36^ The type and concentration of lipid and surfactant, their ratio, time of sonication or agitation, speed are some of the parameters to be optimized to obtain a reproducible method resulting small size nanocarriers. Low dispersion quality is a disadvantage of high shear homogenization and ultrasonication. Dispersion quality of the lipid nanoparticles produced by these techniques is often affected by the presence of microparticles leading to physical instability upon storage.^37^ Metal contaminations from the equipment is the other important problem associated with ultrasonication.^35^

### Phase inversion technique

It is a novel, cost effective and solvent-free approach for the formulation of lipid nanocarriers that involves the phase inversion from o/w to w/o emulsion. It involve two steps.

Step 1 involves mixing of all the ingredients (lipid, surfactant and water) in optimized proportions. The mixture is stirred and temperature is increased at a rate of 4°C to reach up to 85°C from room temperature. Three temperature cycles (85–60–85-60-85°C) are applied to the system to reach phase inversion zone.

Step 2 results an irreversible shock introduced to break the system, due to dilution with cold water (0^o^C). This fast addition of cold water causes formation of nanocapsules. Application of a slow magnetic stirring for 5 minutes avoids particle aggregation. Low energy involvement enables the formation of stable transparent dispersions (smaller than 25 nm), which can be used for encapsulation of numerous bioactive compounds.^38^

### Microfluidizationmethod

The technique involves use of a new, patented mixing technology employing high shear fluid device known as microfluidizer. In this process, the liquid is forced at speed up to 400 m/s through microchannels to an impingement area at high operating pressures. Cavitation and the accompanying shear and impact are accountable for the efficient particle size reduction within the “interaction chamber”. The technique can be utilized on laboratory as well as production scale.^39^

### Membrane contactor technique

Membrane contactor is used to identify membrane systems that are employed to “Keep in contact” two phases. The lipid phase, at a temperature above its melting point is placed in a pressurized vessel. It is allowed to permeate through ceramic membrane pores under applied pressure to form small droplets. The aqueous phase, under continuous stirring, flow tangentially inside the membrane module, and brush away the droplets formed at the pore outlets. Cooling of the preparation to room temperature leads to the formation of lipid particles. Temperature of aqueous and lipid phase, aqueous phase tangential-flow velocity and pressure of lipid phase and membrane pore size are the process parameters affecting size of lipid nanocarriers. The benefits of this new process of membrane emulsification are commercial scalability and control on particle size by fitting optimized parameters.^40^

### Characterization of NLC

Appropriate techniques are required for characterizing physicochemical properties of NLC in order to ensure their performance, product quality and stability. Various evaluation parameters like particle morphology, interfacial properties, drug entrapment efficiency, crystallinity studies etc enlighten the workability of NLCs as drug delivery system.

### Particle size measurement

Particle size of NLC is generally determined by photon correlation spectroscopy (PCS) using Zetasizer which works on Mie theory. Photon correlation spectroscopy (alias dynamic light scattering or quasi-elastic light scattering) is based on the measurement of the fluctuations in scattered light arising from Brownian motion.^41^ It provides the average particle size (z-average) and polydispersity of the system as a measure of the particle size distribution. It characterizes particles of few nanometers to about 3 microns. Laser diffractometer (LD) can characterize a wide range from the nanometer to the micrometer range particles. This evaluation is based on the diffraction pattern depicting particle shape and size (explained by Fraunhofer theory).^1^ In progression, combining Laser diffraction with Polarization intensity differential scattering technology results in the sizing of particles as small as 10 nm.

Data obtained is interpreted by calculating the volume distribution denoted by Dv(10), Dv(50), Dv(90).The parameter Dv(90) indicates the size point upto which 90% of cumulative volume of material in a given sample is contained. Similarly, Dv(50) value gives size point below which 50 % of the sample has the given size. In particle size determination, span is a parameter depicting polydispersity index (PI), i.e. how wide is size distribution of a nanoparticles population. It is the measure of particle homogeneity and its value lies between 0 and 1. A high span value indicates a wide distribution in sizes and a high polydispersity.^42-44^ Theoretically, monodisperse populations indicates PI = 0. Therefore, PI measurement is essential to confirm the narrow size distribution of the particles.^45^

$$SPAN=\frac{Dv(90)-Dv(10)}{Dv(50)}$$

Another technique for particle size classification is by field flow fractionation (FFF) based on the size dependent migration and accumulation profile of particles on application of a perpendicular external field in a laminar flow. The classification into different size fractions can be realized by gravitation (sedimentation FFF), by thermophoresis in temperature gradients (thermal FFF), by electric fields (electrical FFF) or by hydrodynamic fields (cross flow FFF).^46^ Other reported techniques used for particle size determination are dynamic ultramicroscopy, (characterization), ultrasonic spectroscopy, cryogenic transmission electron microscopy (cryo-TEM) analysis, electroacoustic mobility spectroscopy. Determining the particle size is of great importance in nanostructured formulations as the small particle size contributes to a greater interfacial area, which can then provide better drug partitioning and absorption at the skin surface.^47-49^

### Zeta potential

Zeta potential (ZP) is a key factor for evaluation of the stability of nano dispersion. The ZP determination is based on particle electrophoretic mobility in aqueous medium. Zeta Potential characterizes the surface charge and gives the information about long term stability. At higher ZP the particle aggregation is less likely due to electric repulsion while dispersions with lower values tend to coagulate or flocculate, possibly leading to less stability.^50^

Generally the zeta potential of dispersion should be either less than -30 mV or greater than +30 mV for electrostatic stabilization of NLC.^51^ Zeta potential estimation can be done by Laser Doppler electrophoresis, using a Malvern ZetaSizer Nano ZS. By applying an electric field across the sample, particles with a zeta potential will migrate toward the electrode of opposite charge with a velocity relative to the magnitude of the zeta potential. The velocity is measured utilizing the technique of Laser Doppler anemometry, also known as Laser Doppler velocimetry.^52,53^ The frequency shift of an incident laser beam caused by these moving particles is measured as particle mobility, and this is converted to the zeta potential according to the Henry equation. Zeta potential is influenced by factors like electrical conductivity, pH, and the nature of the reagents.^53^

### NLC morphology

Surface morphology of NLC can be observed by transmission and scanning electron microscopy (TEM, SEM), atomic force microscopy (AFM) and PCS. These techniques are tried and true for dimensional and structural characterization of NLCs.^13^

Negative staining, freeze-fracture and vitrification by plunge freezing are different methods of sample preparation for TEM which can furnish different information about the colloidal particles. The sample is put on a gold or copper grid with defined mesh size grid and stained with heavy metal salt solution which provides high contrast in the electron microscope. After drying, the sample is viewed in the electron microscope where the Nanoparticles appear bright against the darker background of the stain.^54^ Dehydration during sample preparation may lead to structural changes, thus original morphology of nano carriers may get altered. Cryo-transmission electron microscopy (cryo-TEM) and cryo-electron tomography (cryo-ET) is robust and indispensable tool to visualize colloidal drug delivery system. The methodology allows investigation of the sample in a vitrified frozen hydrated state, allowing visualization of nanoparticles basically as they exist in solution.^55^ Sometimes use of surfactant in SEM imaging leads to artifacts due to formation of smooth camouflaging coating on particle surface.^56^

AFM is a relatively simple and non-invasive technique that could be abused as a main imaging tool to measure & manipulate morphology and size of lipid nanoparticles. AFM offers an upper edge in characterizing heterogeneous materials and providing high grade data.^57^ Samples for AFM requires fixation by removal of water, which may cause alteration in the status of emulsifier and polymorphic changes in lipids.^46^ The technique does not utilize beams or radiations however a sharp-tipped scanning probe fixed to the free end of a spring like cantilever. Deflection, oscillation or shift in resonance frequency of cantilever motion are used to quantify interaction between the tip and surface of the specimen.^11^

### Entrapment efficiency

It is the influence on the release characteristics that determines the amount of drug loaded in NLC, a prime importance. Entrapment efficiency (Ee) defines the ratio between the weight of entrapped drug and the total weight of drug added to the dispersion. The amount of drug encapsulated per unit weight of the NLC is determined by ultrafiltration-centrifugation method. A known dispersion of NLCs is prepared and centrifugation is performed in a centrifuge tube mounted with an ultrafilter. After appropriate dilution the amount of free drug in supernatant is determined by spectrophotometer.^43^

The entrapment efficiency in the NLCs is calculated by the following equation.

$$Entrapment\;Efficiency\;\left(\%\right)\;=\frac{Wa-Ws}{wa}*100\;\%$$

where, *Wa* is the initial weight of drug used and *Ws* is the amount of drug determined in supernatant after separation of the lipid and aqueous phase.^58^

Loading capacity *Lc* expresses the ratio between the entrapped drug and the total weight of the lipids. It is determined as follows:

$$Lc=\frac{Wa-Ws}{Wa-Ws+Wl}*100\;\%$$

where *Wl* is the weight of lipid added in the formulation.^58^

Liquid lipids create a disordered crystal matrix and consequently more space is available for loading large amount of drug, hence enhancing entrapment efficiency. *Ee* and *Lc* are dependent on several parameters, such as the lipophilic properties of the active pharmaceutical ingredient, the screening of the most appropriate lipid composition/ratio (solid/liquid lipids) and surfactant combination, as well as the production procedure used.^59^

### Crystallinity and polymorphism

The characterization of the crystallinity of the NLC components is important as the lipid matrix as well as the loaded drug may undergo a polymorphic transitional change leading to a possible undesirable drug leakage during storage.^60^ The status of crystallinity of a particle also influences the encapsulation efficiency & release rates.^61^ An increment in thermodynamic stability and lipid packing density, while a decrease in drug incorporation rate is observed in the following order^46^: Supercooled melt > alpha modification > beta’ modification > beta modification

To investigate crystallinity and polymorphic status, differential scanning calorimetry (DSC) and X-ray diffraction (XRD) experiments are performed.

DSC gives the heat lost or gained because of physical or chemical changes within a sample as a function of the temperature. DSC measurements reveal the status of lipid, melting and crystallization behavior of solid lipids in nanostructures.^62,63^ DSC analysis is carried out on pure drug, pure lipids and nanoparticles. DSC characterization can illuminate NLC structure through the mixing behavior of solid lipids with liquid lipids.^64^ The breakdown and fusion of the crystal lattice by heating or cooling the sample furnish exclusive information of polymorphism, crystal ordering, eutectic mixtures, glass transition processes and drug lipid interactions.^64,65^ Recrystallization index (RI) is a parameter to perform comparative study of the crystallinity between the developed formulations. It can be calculated from the following formula:

$$RI=\frac{\text{ΔHNLC}}{\text{ΔH bulk X Concentration of lipid}}\text{X }100$$

where ΔH NLC =Melting enthalpy of 1 g NLC suspension,

ΔH bulk = Melting enthalpy of 1 g bulk lipid,

ΔH is given in J/g and the concentration is given by the percentage of lipid phase.^66^

Gonullu et al determined Crystallization index (CI %) to determine the crystalline state of the drug in formulations.

$$CI=\;\frac{Ms\;}{Mp\gamma }\times 100$$

where, Ms = Melting enthalpy (J g–1) of lipid nanoparticles, Mp = Melting enthalpy (J g–1) of pure solid lipid, and ϓ represents solid lipid concentration (%) in nanoparticle dispersion.^62^

An increase in amount of liquid lipid lowers the crystallinity and increases the defects in highly ordered structure of NLCs. The principle behind performing DSC rests on different enthalpy and melting point for different lipid changes. NLCs having a smaller size, therefore a higher surface area and employing more surfactants showed a decline in enthalpy and melting point of lipids.^10^

XRD analysis is another useful technique to reveal polymorphic structural changes of compounds In XRD, the monochromatic beam of X-ray is diffracted at angles as per the type and arrangement of the atoms and space between the planes in the crystals.^60^ Lipids has the ability to aggregate in a variety of ways giving rise to different polymorphic forms. This can be in form of micelles, lamellar phase, tubular arrangement or cubic phases. Wide angle and small angle X ray scattering techniques (WAXS, SAXS) are utilized to explore the layer arrangements, crystal structure, phase & polymorphic behavior of lipid and drug molecules. It also gives an idea regarding length of the short and long spacing of lipid lattice^46^ and localization of drug in it.^67^

### Surface tension measurement

An increase in concentration of emulsifier lowers the interfacial tension of system due to the emulsification process. Surface tension of the lipid nanoparticles is often measured by the Wilhelmy plate method. The measurement of the contact angle is another method for detecting surface tension of the nanoparticulate systems.^11^

Kibron instrument is a high precision, easy to use torsion balance instrument to measure surface tension of NLC. It employs the combination of maximum pull force technique and a unique ultrasensitive microbalance with Kibron’s proprietary sensor. The instrument is at par in the technique of determining surface tension in comparison to platinum Wilhelmy plates or Du Noüy ring.^68^

### Evaluation of additional colloidal structures

In various cases, it has been observed that other colloidal structures like micelles, mixed micelles, liposomes, supercooled melts, accompany lipid nanoparticles. Evaluating these colloidal structures is quite a difficult task due to their size similarity, low resolution of PCS to image multimodal size distribution. Sample preparation may alter the equilibrium of the complex colloidal system. It may also require dilution of the dispersion with water which can lead to removal of surface active agent from nanoparticle surface. On this account technique like nuclear magnetic resonance (NMR) and electron spin resonance (ESR) are extremely useful tools for the simultaneous detection of colloidal structures and no sample preparation. These non-destructive methods are fruitful for examining dynamic phenomena and the presence of the nano oil compartments in colloidal lipid dispersions.^69^

#### Magnetic resonance investigation

NMR spectroscopy (1HNMR) and ESR are employed to enquire about structure of lipid nanoparticles and loaded drug.^70^ The spectroscopy is used to judge mobility and interaction of oil molecules with solid lipid.This non-invasive technique permits a simple and rapid detection by virtue of width of signal. Proton relaxation times are associated with NMR line width. Mobile oil molecules will give small line widths with high amplitudes, whereas molecules with confined mobility give weak amplitudes and broad signals. Therefore, the technique is apt to evaluate the extent of immobilization.^46,71^

#### Negative staining

Negative staining is used to increase the electron opacity of the surrounding field. This technique uses salts of various heavy metals to determine the co-presence of colloidal lipid particles in dispersion by TEM. The test sample placed on a metal mesh grid is stained with solutions of heavy metal salts such as uranyl acetate, ammonium molybdate, sodium silicotungstate or sodium phosphotungstate that enhances the contrast. Sections are completely dried before attempting microscopic imaging. Presence of quick dried and stained artefacts makes it tedious to differentiate them from actual colloidal particles. The optical micrographs generated are of low resolution due to grain size stain. Negative staining electron microscopy may give three dimensional projections but problem may arise due to insufficient views on account of attachment of sample to the continuous carbon support in a preferred orientation.^10,72^

#### Electron spin resonance (ESR)

ESR investigation requires paramagnetic spin probes and no sample preparation. The spectra furnish information about molecular mobility and polarity inside the drug delivery system. This technique gives direct and noninvasive evaluation of the distribution of the spin probe between the aqueous and the lipid phases.^46^ ESR spectra gives signal for paramagnetic lipids. This tool helps to understand the characteristic properties of nano compartments in lipid carriers.^71^

#### Raman spectroscopy

It is an excellent tool to answer many questions about structural properties of lipids. The technique requires no preliminary steps and permit detections in the presence of water.^63^ Fourier-transform infrared spectroscopy and Raman studies are conducted to detect drug-lipid interaction. Raman spectroscopy study excited molecular vibrations produced by a laser beam. The technique is used to map chemical and structural changes in molecules from their transition between two vibrational states. Raman spectroscopy is a useful tool to study packing, conformation and any changes in lipid chain arrangement after loading oils.^70^

### Structure of skin barrier

Skin, the largest multilayered organ of the body, with a surface area of 1.7-2.0 m^2^, is composed of several layers: stratum corneum (SC), the viable epidermis, the dermis and lower layers of adipose tissue. The SC, the superficial layer is the main roadblock of the skin. The SC comprises of corneocytes encompassed by lipid domains like long chain ceramides, free fatty acids, cholesterol, sterols and phospholoipds.^41^ Corneocytes are covered by a cornified cell envelope (insoluble) which acts as a critical skin barrier formed by covalent cross-linking of component proteins such as involucrin, loricrin, and the small proline-rich protein.^73^ The corneocytes are inserted in lipid lamellar locales, which are orientated parallel to the corneocyte surface. Since most drugs administered via skin dependably need to permeate along the tortuous pathway in these lipid locales, the organization and composition of the lipid component is viewed as imperative for the skin hindrance.^74,75^ It is therefore, the lipids play an essential job in the skin hindrance, which makes their shared course of action in the lamellar areas, a key procedure in the development of the skin obstruction. Low pH of skin, presence of enzymes, skin hydration, diseased state and the transcutaneous concentration gradient are the other factors conceptualizing the barrier property of skin. Percutaneous absorption of topically applied agents also influenced by molecular weight, partition coefficient, formulation design, presence of penetration enhancer.^76^

### Beneficial role of NLC in transdermal drug delivery

Transdermal drug delivery system is an established system since ages to attain diverse therapeutic objectives on different structural levels of skin (e.g. surface, epidermis, dermis and hypodermis). However, several problems associated with the conventional topical preparations have come in picture e.g. impermeability to skin barrier, limited efficacy and high frequency of application. The current focus of the researcher is quite related towards exploiting NLC for topical and dermal application, both in pharmaceutical as well as cosmetic sector. NLC are composed of biologically active and biodegradable lipids that show less toxicity and offer many favorable attributes such as adhesiveness, occlusion, skin hydration, lubrication, smoothness, emolliency, skin penetration enhancement, modified release, improvement of formulation appearance providing a whitening effect and offering protection of actives against degradation.^2^ The key advantageous features offered by NLC that makes its role superior in transdermal drug delivery are as follows:

### Skin permeation

Umpteen scientific literatures has stated about capability of NLC to control the rate of drug penetration into the skin, which limits the unwanted active absorption into blood circulation.^77^

**Figure 2:**
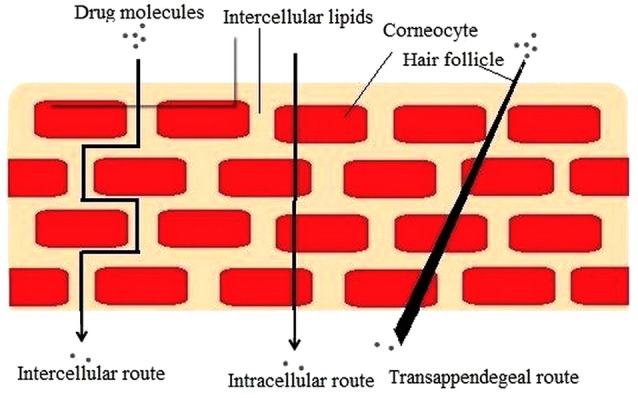
Routes of drug penetration across the stratum corneum.^76^

The smaller size of NLCs ensures a nearby contact with SC and can enhance the skin penetration of active compound. Investigators have reached consensus on the routes of nanoparticle penetration (Figure 2) through the skin. Proposed mechanisms for enriched permeation of the particles via the stratum corneum pursued by diffusion of drug are as follows:

1. Unblemished medication loaded vesicle entrance into the diverse layers of the skin.
2. Lipid vesicles acting as penetration enhancers via their skin lipid-fluidizing and modifying property.
3. Induction of nanostructure lipid carrier and skin drug exchange after mingling of carrier lipids with cellular skin lipids.
4. Appendageal route including the hair follicles (HF), pilosebaceous, and sweat gland pores.^57^

Attributable to the hair follicle speaks to an invagination of the epidermis expanding profound into the dermis, which results in a more noteworthy genuine region for drug absorption. The hair follicle has turned into the most essential penetration pathway for nanoparticles among the appendageal routes. Besides, the hair follicles speak to an effective store for topically administered nanoparticles, which are normally extended deep into the tissue up to 2000 μm. Both permeation enhancement and sustained release are attainable by virtue of larger storage volume of hair follicle casts. Drug particle accumulate into the follicular casts followed by diffusion of drug from the nanocarriers into the skin.^78^

Skin penetration of NLCs depend on their composition, and their physicochemical properties like size, aggregation, charge on particle surface, hydrophobicity, solubility of particles in the skin, solubilizing properties of particles towards the skin lipids, and whether particle possess film forming ability.^73^

### Transepidermal water loss (TEWL)

TEWL is the amount of water that passively evaporates through skin to the external environment due to water vapor pressure gradient on both sides of the skin barrier and is used to characterize skin barrier function.^79^ TEWL loss measurements indicate damaged and weakened barrier function of the stratum corneum. An increase in TEWL marks a disruption of the stratum corneum and depletion of intercellular lipids.^41^ Topical application of NLC depletes TEWL due to enhanced skin occlusion leading to skin hydration. It was observed that application of NLCs assisted to reduce moisture loss from the skin in comparison to the untreated skin. This owes to the ultrafine size of lipid nanocarriers actuating high surface area, and improved adhesive properties. The lipid particles forms a uniform compact film over the skin, hence inhibiting water evaporation from the skin.^80^ Smaller the size of the particles, the reduced water evaporation from the skin.^41^

It was found that the presence of a highly crystalline lipid in formulation decreases moisture loss from the stratum corneum effectively.^81^ The presence of emollients (lecithin, propylene glycol) in NLC formulation helps to substitute for the depleted natural lipids present in the space between corneocytes in the stratum corneum to prevent excessive TEWL.^82^

### Skin occlusion

**Figure 3:**
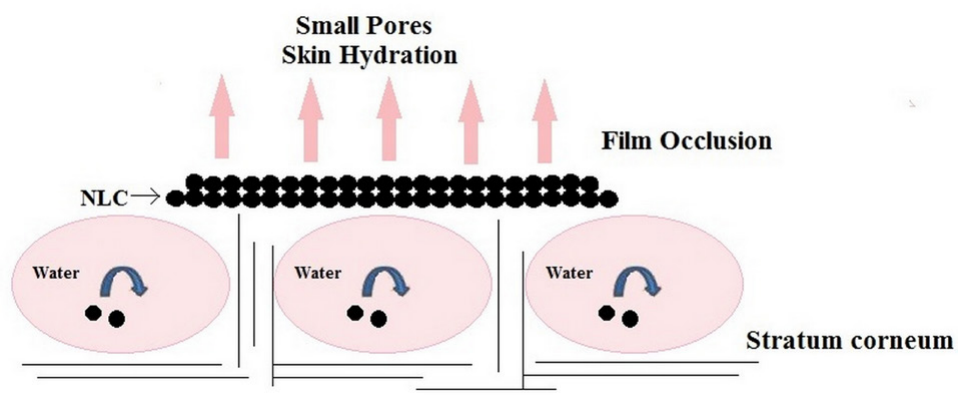
Occlusion effect of NLC.^11^

In general, lipid nanocarriers have peculiar epidermal occlusive characteristics which by inhibiting water evaporation could enhance bioactive penetration into the stratum corneum. Nanoparticles were found to be 15 times more occlusive than microparticles. Müller et al mentioned NLC as “invisible, penetration enhancing occlusive plastic foil.”^83^ Scope of NLC occlusive feature depends on the following factors:

- Particle size: Small particle size of the nanocarriers diminishes water evaporation from the skin. Occlusion factor of lipid micro particles of >1 µm diameter was found to be 10%, whereas lipid nanoparticles of 200 nm size showed 50% occlusion.^84^ Wissing et al also depicts that formation of a monolayer film on skin require 4 mg of topical product with 4% lipid nanoparticles of approx size 200 nm.^81^ This occlusive film as shown in Figure 3, being hydrophobic prevents dehydration of the skin, retards the penetration of UV filters in sunscreens and other whitening effects, lubrication/emolliency and control release properties.^37^

1. Crystallinity and concentration of lipids: High concentration of lipid (50%-60%) present in NLC formulation act as occlusive agent and is responsible for retaining the moisture in the stratum corneum.^41^ It was found that high occlusivity can be attained with low melting lipids and highly crystalline particles. Formulation having particles size less than 400 nm and at least 35% lipid of high crystallinity was found to be most effective. An increase in oil content in NLC formula causes a decrement in occlusive factor.^81^ Supercooled melts (noncrystalline nanoparticles) have no occlusive properties.^61^ An enhanced skin hydration effect showed up due to the occlusive nature of NLCs.^84^

### Skin hydration and elasticity

Usually the human skin sustains a water content of 10%–20% of tissue dry weight. The surface lipid content and moisture state play an important role in the frictional properties of the skin.^41^ Adequate hydration of skin slows down the signs of aging and protects from environmental damage. NLC fabricated from biocompatible and physiological lipids hold fast to the skin and produce an occlusive action expanding skin hydration. Lipid nanoparticle also retard moisture loss, hence loosen the corneocyte packing. The expanded intercorneocyte gap in turn leads to enhanced drug penetration.^85^ NLCs could extend this superior hydration attribute due to its ultrafine size. Ascribed to small size lipid nanoparticles cling to lipid film of stratum corneum (hydrophobic interactions). The adsorbed film reconditions damaged skin and thus restores a protective thin film.^86^ Owing to less particle size, the dimensions of the capillary channels of nanometer pores will be much smaller; thus, the hydrodynamic evaporation of water will decrease.^83,87^

Loo et al depicted a high concentration of lipids in NLCs increase hydration more effectively as a consequence of occlusive layer. It is because of sliding of additional lipids into the intercellular space of the skin that assist in less TEWL.^82^ Presence of humectants in formulation has an additional effect on hydration of SC by attracting moisture from dermis and air. Tichota et al formulated argan oil NLC-based hydrogel manifesting a synergistic effect on skin hydration (NLC occlusion plus argan oil hydration).^88^

Skin hydration can be measured using a corneometer. The instrument works on the mechanism of measuring capacitance or conductance of a dielectric medium. As the hydration level of stratum corneum increases, its dielectric property alters. The method is simple, quick and hardly affected by skin residues.^82^ It has been observed that skin hydration gets amplified with increased con­centration of lipids.^41^ Skin microrelief is used to evaluate skin-hydration efficacy of cosmetics, and could be assessed by measuring the parameters of roughness, scaling, smoothing, and wrinkling.^88^ The moisturisation effect of a product leads to the increase in skin hydration, which contributes to wrinkle smoothing and enhancing the penetration of bioactives into specific skin layers.^82^ Any alteration in the hydration level of the skin are measured in arbitrary units.

### Stability of bioactives

One of the most important features of lipid nanoparticles is the ability to retard the chemical degradation of actives by photochemical, hydrolytic and oxidative pathways. Chemical stability of drug molecule stoutly depends on solid lipid matrix of lipid nanoparticles. Researchers remarked that the chemical stability of NLC fabricated with less crystalline solid lipid and lattice imperfection were improved due to increased drug arrangement within the lipid matrix. Solid state minimizes the exchange of actives with water phase (diffusional law by Einstein).^89^ Moreover, the structure and good chemical stability of lipid itself must be reviewed. Hence, the selection of most suitable lipid during preformulation studies is quite important. It has been noted in research that actives that are incorporated in the imperfections at the less crystalline lipid matrix of NLC provide prolonged physical stability.^65^ Table 3 displays a brief literature of drugs incorporated in NLC to increase stability.

**Table 3:** Literature review of drugs incorporated into NLC to enhance stability

**Name of drug**	**Method of preparing NLC**	**Excipients used**	**Details**	**Ref.**
Idebenone (IDB)	Modified high-shear homogenization and ultrasound method	Glyceryl palmito-stearate, Medium chain fatty acid triglycerides	1. Medium chain fatty acid triglycerides as liquid lipid, possesses a higher solubility for IDB than solid lipids and is incorporated into the core of a solid lipid. The drug is probably in the liquid lipid which in turn is surrounded by the solid lipid. 2. This provides some degree of mobility to the drug which contributes to stability even when the solid lipid undergoes polymorphic changes. A reduction in IDB expulsion from the disordered nanocompartments within the solid matrix avoids IDB degradation.	90
Coenzyme Q10 (CoQ10)	Hot high pressure homogenization technique	O/020G, O/100G, Glycerin monostearate, Glyceride, Span 20	1. CoQ10 existed as amorphous form in the NLC-based formulation and showed considerably enhanced photo-stability compared with CoQ10 itself. 2. CoQ10-NLC possessed negatively charge and highly stable dispersion with nanoscale diameter when they were dispersed in water.	57
Alpha-lipoic acid (ALA)	Hot high pressure homogenization technique	Glycerin monostearate, Glyceryl triacetate	1. About 88.5% of the initial ALA in NLC system remained after 120 days under the same conditions, while the retention of free ALA was only 0.7% under natural daylight irradiation. 2. Encapsulation of ALA inside the nanodroplet provided better photo-stability protection.	91
Retinyl Palmitate (RP)	Ultrasonication method	Stearic acid, cetyl palmitate, Virgin coconut oil	1, NLC was found to be superior formulation to protect RP against stressed conditions of light, temperature and hydrolytic degradation. 2. During storage for 28 d, retinyl palmitate in NLC degraded only about 15% as compared to microemulsion where 50% drug degraded.	92
Lutein	High pressure homogenization.	Glyceryl tripalmitate, Carnauba wax Miglyol® 812	1. NLC improved the lutein thermostability and photostability 10 times more than the free form. 2. Only 6%-8% degradation was observed after irradiation. 3. Carnauba wax (solid lipid) acts as a molecular sunscreen hence contributing to the excellent photo stabilizing effect.	93
Ascorbyl palmitate (AP)	High pressure homogenization technique	Imwitor® 900 (glyceryl monostearate), Labrafil® M1944 (apricot kernel oil polyethylene glycol-6 ester), Hydrine® (PEG-2 stearate), Apifil® (nonionic hydrophilic white beeswax)	1. Choice of surfactant was one of the important factors to increase the stability of AP aligned at the interface. 2. Surfactants get adsorb on surfaces or interfaces of the system and modify the surface or interfacial free energy. 3. It was also found that increasing the drug concentration up to saturation solubility in the melted lipid leads to the ‘drug enrich core’ model. 4. Higher AP loading leads to encapsulation of drug in lipid matrix compared to lower drug loading. In this event AP molecules could be protected from oxygen molecules.	65
Phenylethyl resorcinol (PR)	Hot high-pressure homogenization method.	Glycerin monostearate, diglycerides, BehenylAlcohol LL- octyl and decyl glycerate	1. In 90 days’ storage, 88.6 ± 2.8% of PR remained unchanged in PR-NLC under natural daylight. 2. Incorporation of PR into NLC could give greater chemical stability, particularly photo-stability, during storage under natural light exposure as compared to that of free PR.	94

### Prolonged effect

Release profile from lipid nanoparticles is drastically influenced by type of lipid (solid or oil) used to formulate the vehicle, concentration of surfactant (or surfactants), solubility and concentration of active in the lipid matrix and method of formulating NLC.^77^ NLC are produced by controlled mixing of solid lipids with spatially incompatible liquid lipids leading to special nanostructures with improved drug incorporation and release properties.Table 4 present a review of work done for extending drug release using NLC.

**Table 4:** Literature review of drugs incorporated into NLC for prolonged release

**Name of the drug**	**Method of Preparation**	**Objective of Prolonged release**	**Details**	**Ref.**
Aceclofenac	Melt-emulsification and low-temperature solidification method Ultrasonication method or high-speed homogenizer method	To formulate a controlled-release drug delivery system for a prolonged period to satisfy the goals of the treatment of arthritis like reducing pain and inflammation	1. Oleic acid played an important role in the release of aceclofenac from the NLC dispersion. 2. Liquid lipid being located in the outer shell of the nanoparticles, leading to a drug-enriched shell that is related to burst release at the initial stage. 3. No significant effect of the method of preparation on the release of drug.	42
Amoitone B	Emulsion-evaporation and low temperature-solidification technology	To construct an effective delivery system for Amoitone B to realize sustained release, thus prolong drug circulation time in body and improve the bioavailability.	1. When contacting the release solution, the superficial drug was freed quickly, leading to the burst release in the initial stage. 2. With the degradation and erosion of lipid matrix, the drug incorporated into nanoparticles core was released in a prolonged way.	95
Oridonin	Emulsion-evaporation and low temperature-solidification technique	To prepare oridonin-loaded NLC, in which the Nanoparticles can be obtained in mild conditions easily without the need of any special equipment and the sustained drug release can also be achieved.	1. During emulsification, at first, most of the drug was dispersed in lipid droplets due to low solubility in water. 2. With increasing temperature and the existence of surfactant which increased drug solubility in the water, drug was migrated from the lipid phase to water phase. 3. During the cooling of the produced O/W nanoemulsion, a re-partitioning of the drug into the lipid phase occurred. 4. When reaching the recrystallization temperature of the lipid, the solid lipid core was rapidly solidified to form a solid lipid core in which liquid lipid was randomly distributed.	96

### Skin targeting

Targeted drug delivery to the epidermal layer could display immense benefits over systemic administration on countless therapies. For the treatment of skin ailments like acne, a fungus infection or hair fall; the efficacy of topical drug is speculated by their potential to reach the desired site of action (specific skin layers) and remain there in therapeutic effective concentration for the appropriate time. Poor therapeutic effects & adverse reactions exhibited by conventional topical carriers can be conquered by formulating dermal NLC.^22^ The strategy to target epidermis layer is favorable in respect of safety and as well as a rich network of antigen-presenting cells in epidermis eliciting a higher immune response.^78^

Researchers are yet to be in unison about the fact that which particle property would result in superior skin targeting. It has been found that drug characteristics like size of particulate carrier, surface charge and composition material affect skin permeation and pharmacodynamics of encapsulated drugs. Degree of drug binding with plasma protein affect the epidermal targeting and penetration of particle deep into HF, on account of the capillary network around this structure, predominantly in the growth phase. Following accumulation in HF, nanoparticles can diffuse rapidly and continuously into the dermis or subcutaneous region enhancing epidermal targeting as well as local delivery of drug.^78^

**Figure 4:**
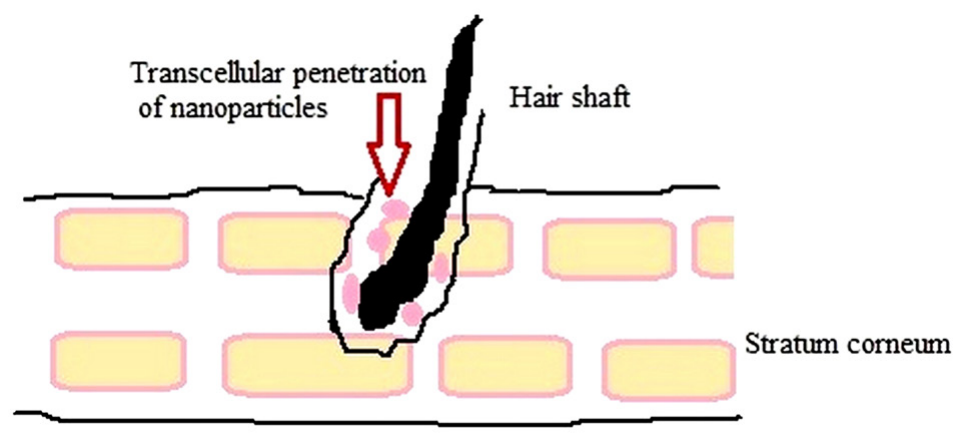
Transfollicular Penetration of Nanoparticles.^11^

It was observed that adjusting the size of particulate carriers enables an intra- or transfollicular penetration **(**Figure 4) and drug delivery. Particles of different sizes and structures accumulate in the hair follicle openings and penetrate along the follicular duct, when applied onto the skin. Particle-based drug delivery systems can be used to target specific regions within the follicular duct as penetration depths are counted upon the size of the particles and on the hair follicle type. The motion of the hair may likewise add to the penetration of nanoparticles of a size of 20 nm.^97^ Lipids that resemble the composition of sebum may likewise advance follicular penetration as a high sebum/water partition coefficient is a basic characteristic for transport into HF. Epidermal targeting may also be assumed due to a dissolution or erosion of lipid particles in sebum. Manipulating surface charge can also be used as an approach for targeting.^78^

#### Drug molecules incorporated into NLC for skin targeting

Podophyllotoxin, oxiconazole nitrate, clobetasol, curcumin, tretinoin, minoxidil, finasteride, diphencyprone, terbinafine hydrochloride.

### Regulatory perspective

One of the significant grounds for the wide acceptance and commercial success of NLC is that these carriers present minor regulatory obstacles. The nanostructure lipid carriers employ lipids and emulsifiers with physiological, nontoxic, biodegradable and compatible profiles. All the ingredients used are acknowledged GRAS status by regulatory authorities or already have been accepted for encapsulating pharmaceutical or food active compounds. Still in any case, it is essential to use all the ingredients in safe and accepted range. Most of them are obtained or consist of ingredients occurring naturally in the human body i.e. fatty acid & glycerol. These are known to be well tolerated and reduce cytotoxic or adverse drug reaction.^22,77,83^

### Patent status of NLC

Over the past decade, nanostructure lipid carriers have been exploited for formulating a number of transdermal and cosmetic agents. Various patents have been granted for nano-lipid formulations. Table 5 presents a brief review of patents on the subject of original invention in the nanostructure lipid carriers.

**Table 5:** Patent status of NLC

**Patent Name**	**Patent Number**	**Inventors**	**Publication Date**	**Reference**
Novel nano-lipid carrier for injection embodying paclitaxel series substances and preparation method thereof	CN101366697A	Liu et al	18/2/2009	98
Nanostructured lipid carriers containing riluzole and pharmaceutical formulations containing said particles	EP20070764871	Bondi et al	20/10/2010	99
Use of nano structured lipid carrier drug feeding system	CN101129335A	Jian et al	22/9/2010	100
Composite anti-screening agent nanostructured lipid carrier and preparation method thereof	CN102688152A	Qiang et al	26/9/2012	101
Lipid nanoparticle capsules	US 2013/0017239 A1	Petit et al	17/1/2013	102
Bionic lovastatin nano-structured lipid carrier and preparation method thereof	CN102935077A	Jianping et al	20/2/2013	103
Coenzyme Q nanostructured lipid carrier and preparation method thereof	CN101658468A	Summer et al	6/3/2013	104
Nanoparticle formulations for skin delivery	US8715736B2	Sachdeva et al	06/05/2014	105
A composition for treating leukemia	WO2014123406A1	Abdullah et al	14/8/2014	106
A method for producing nanolipid formulation for skin care and/or repair and a nanolipid formulation of the same	WO2015105407A1	Ujang et al	16/7/2015	107
Lipid nanoparticles for wound healing	EP2821077A1	Lafuente et al	7/01/2015	108
Preparation of nanostructured lipid carriers (NLC) method and products made	CN102283809B	Ismail et al	14/12/2016	109
Nano-structured lipid carrier comprising α-tocopherol and preparing method thereof	KR101777616B1	Geun et al	13/9/2017	110
Nanostructured carriers for guided and targeted on-demand substance delivery	US Patent Application 20170119891	Lal et al	4/5/2017	111

## Conclusion and Future Perspective

NLC provide a large influence to the enormous bars that are a prerequisite in formulating a stable drug delivery system. The application of nanocarriers in transdermal drug delivery has proclaimed a new domain in the drug delivery. NLCs are chemically and physically stable system with improved drug incorporation and increased bioavailability. The increasing interest of industry in lipid carrier system has been remarkably expanding the advancements in latest years. At present more than 30 commercial NLC formulations are accessible in the market containing drug and cosmetics ingredient. NLC as smarter generation of lipid nanoparticles are promising candidates to provide skin targeting along with occlusive effect and prolonged release. Lipid nanocarriers are seeking industrial attention due to qualified and validated scale up of technology, GRAS status of excipients and easy large scale production. Future NLC formulations can bring more prosperity to the lipid carrier system because of its umpteen superior characteristics over first generation systems. Future concern also involves assessment of toxicity and health hazard associated with nanostructures. More research in preclinical and clinical investigation will pave the way to success for nano-lipid structures. Achievement in this field can be conceived if pharmaceutical industry picks up the academia research to style this carrier system for various therapeutic and cosmetic agents.

## Ethical Issues

Not applicable.

## Conflict of Interest

The authors declare that this article content has no conflict of interest.
